# Efficacy of Deep Cervical Flexor Muscle Training on Neck Pain, Functional Disability, and Muscle Endurance in School Teachers: A Clinical Trial

**DOI:** 10.1155/2021/7190808

**Published:** 2021-01-13

**Authors:** Zaheen A. Iqbal, Ahmad H. Alghadir, Shahnawaz Anwer

**Affiliations:** ^1^Rehabilitation Research Chair, College of Applied Medical Sciences, King Saud University, Riyadh, Saudi Arabia; ^2^Department of Health and Physical Education, The Education University of Hong Kong, Tai Po, Hong Kong; ^3^Department of Building and Real Estate, Hong Kong Polytechnic University, Kowloon, Hong Kong Special Administrative Region, China

## Abstract

**Background:**

Neck pain (NP) is a common work-related disorder, with high prevalence in the profession of teaching. The daily duties of a school teacher involve head-down postures while reading and writing, which expose them to the risk of developing NP. Deep cervical flexor (DCF) muscles have been reported to have lower endurance in patients with cervical impairment, which has additionally been associated with disability. There is limited evidence regarding the efficacy of training of DCF muscles in occupational NP. The objective of this study was to investigate the effects of DCF muscle training on pain, muscle endurance, and functional disability using pressure biofeedback in school teachers with NP.

**Methods:**

Sixty-five teachers (age, 25-45 years) with more than 5 years of teaching experience participated in this study. They were randomly divided into two groups: the experimental (E) and control (C) groups. In the E group, the subjects underwent DCF muscle training using pressure biofeedback in addition to conventional exercises for neck pain, while those in the C group underwent conventional exercises only. Pain, muscle endurance, and disability were measured at day 0 (before the treatment) and days 14 and 42 after the treatment. Endurance of DCF muscles was measured by the craniocervical flexion test using pressure biofeedback, pain intensity was measured using the numeric pain rating scale, and functional disability was assessed using the neck disability index questionnaire. This study was performed in accordance with CONSORT guidelines.

**Results:**

On day 0, there were no significant differences in the age, pain, muscle endurance, and disability levels between the groups. After initiating the intervention, although there were improvements in both groups, there was a statistically significant improvement in muscle endurance, pain, and disability in subjects who received additional training with pressure biofeedback.

**Conclusions:**

Besides increasing muscle endurance, specific training of DCF muscles in addition to conventional exercises can improve neck pain and functional disability. These results should be further correlated clinically. A dedicated time for exercises at school could help prevent the development of NP in teachers. This trial is registered with ClinicalTrials.gov NCT03537300 May 24, 2018 (retrospectively registered).

## 1. Introduction

Neck pain (NP) affects approximately 70% of the world population at some point in life [1]. It is reported to be one of the most common occupational disorders, and the physical nature of the job is a risk factor for its development [2]. “Job” as a risk factor for the development of NP includes multiple aspects and job ergonomics; bending the head forward is only one of them [3]. Other risk factors that have been identified as potential causes for the development of neck and upper extremity pain include work-related psychosocial aspects [4]. These include various factors associated with the job such as the demands, work situation, and lack of support from coworkers [5, 6].

Teaching is one such occupation where the prevalence of NP is high with reports suggesting that at least 69% of high school teachers suffer from NP [7], with a lifetime prevalence of 47% [8]. The daily duties of school teachers involve a lot of time in the head-down posture during reading and writing [9], which exposes them to the risk of developing NP. High work load, lower colleague support, and high anxiety levels are other factors that have been reported to be responsible for the development of NP in teachers [10, 11]. Furthermore, NP results in disability that affects social performance and mental wellbeing, further leading to a poor quality of life [9, 12]. With the advances in information technology, traditional methods of teaching have been replaced with modern methods such as the internet, projectors, computers, and smart classes [10], which have increased the number of hours a teacher has to use the computer. Longer duration of computer use has been associated with NP [13]. A static posture for prolonged duration has been reported to cause NP in persons who use the computer for long durations, leading to static muscle tension in the neck region [14]. Although various studies have demonstrated a high prevalence of NP in teachers, to the best of our knowledge, there are no interventional studies that have demonstrated improvements in pain and associated disabilities in this population.

Deep cervical flexor (DCF) muscles have an important postural function in supporting the cervical lordosis [15]. Previous studies on cervical impairment have reported lower endurance in DCF muscles as the cause of NP, which causes muscular insufficiency [16, 17]. In addition to impaired activation, poor endurance has also been demonstrated in DCF muscles in such cases [18]. Such weakening of the anterior cervical flexor muscles can affect the head and neck posture [19–22]. Craniocervical flexion (CCF) has been reported to isolate DCF muscle activation [23, 24], and Jull et al. have endorsed a specific exercise protocol that involves static contraction of DCF muscles at the submaximal level to improve their function [10, 25]. Although restoration of DCF function has been recommended clinically in the management of NP [26, 27], there is only limited evidence on its efficacy in occupational NP.

The objective of this study was to investigate the effects of DCF muscle training using pressure biofeedback on pain, muscle endurance, and functional disability in teachers with NP. We hypothesized that DCF muscle training with pressure biofeedback in addition to conventional exercises shall yield better results by decreasing pain and disability and increasing endurance.

## 2. Materials and Methods

### 2.1. Subjects

Sixty-five school teachers, aged between 25 and 45 years, with teaching experience of more than 5 years were invited to participate in this study. The inclusion criteria included a chronic NP score of more than 5 on the numeric pain rating scale (NPRS), mild-to-moderate disability on neck disability index (NDI), and poor results on the CCF test. The exclusion criteria included the presence of any neurological signs or symptoms, history of spinal surgery, and any ongoing treatment for the pain. The subjects were informed about the aims and nature of this study, and their informed consent was obtained. This study was approved by the ethical committee of the institutional review board (IRB). This study was conducted according to the CONSORT guidelines.

### 2.2. Study Design

This study was a randomized controlled two-arm interventional clinical trial. A convenience sampling method was used to collect the sample. The subjects were randomly divided into two groups: the experimental (E) group and the control (C) group, using the lottery method by the principal investigator, who assigned unique numbers to each subject on a paper and kept it in a box. One paper was taken out, and the number written on it was used to allocate the groups. This research design study was adapted from a similar research published earlier [10].

### 2.3. Procedure

Subjects in both groups received conventional exercises for NP. In the E group, the subjects also underwent DCF muscle training using pressure biofeedback. Pain, muscle endurance, and disability were measured at day 0 (before initiation of treatment) and days 14 and 42 after initiation of treatment.

The exercise session and measurements were conducted by a trained therapist, who was blinded to the treatment allocation. Subjects were not blinded to the treatment group allocation and were instructed not to receive any other treatment during the study period.

### 2.4. Outcome Measures

Pain intensity was measured using NPRS. The subjects rated their pain on the 0-10 rating scale, where 0 implied no pain and 10 implied the worst possible pain [28].

Endurance of DCF muscles was measured by the CCF test using a pressure biofeedback instrument (Stabilizer TM, Chattanooga Group, Inc., Chattanooga, TN). During the CCF test [27, 29], the subject lay in the crook lying position and the pressure biofeedback instrument was placed under the neutral cervical spine below the occiput and inflated up to 20 mmHg. CCF (gentle head nodding of head as if saying “yes”) movements were demonstrated during the test. Substitution with superficial neck flexors, head retraction, or head lift was discouraged. The subjects performed this movement at 5 different pressure levels, i.e., 22, 24, 26, 28, and 30 mmHg, with 30 s rest between each repetition. Each level was supposed to be held for 10 s, and the test was terminated if they were unable to hold the position for 10 s at any level or if the maximum level was achieved (30 mmHg). This value was used to create an index to score the muscle endurance out of a total of 100, and the minimum requirement for satisfactory endurance was 26 mmHg as described by Grant et al. [30, 31].

Functional disability was assessed using the NDI questionnaire. The subjects answered multiple-choice questions, and their score was interpreted on a range of 0-50, 0 being no disability and 50 indicating complete disability [32].

### 2.5. Intervention

Conventional exercises [23, 25] included stretching of the sternocleidomastoid, upper trapezius, levator scapulae, suboccipitalis, and pectoral muscles. It also included nonspecific strengthening of the neck flexor muscles. Each session comprised of each exercise that included 10 repetitions, each held for 10 s with rest of 2 minutes between sets. The sessions were conducted for 6 weeks (42 days), 4 days a week. Each session did not exceed 20 minutes.

DCF muscle training using pressure biofeedback was conducted as described by Jull et al. [23, 24]. Subjects lay in the crook lying position. The pressure biofeedback unit was placed below the occiput and inflated up to a baseline pressure of 20 mmHg. The subjects were instructed to perform head-nodding action to progressively target 5 pressure levels. This included 3 sets in a session with 10 repetitions each with 2 minutes of rest between sets and 5 days a week for 6 weeks (42 days).

### 2.6. Statistics

The data were analyzed using SPSS 15.0 (SPSS Inc., Chicago, IL). The pain, endurance, and disability levels were compared at day 0 and days 14 and 42 after the treatment. Normality was analyzed using the Kolmogorov-Smirnov test. Nonparametric repeated measures analysis of variance (Friedman test) was used to compare the intragroup differences. The intergroup differences were compared with nonparametric analysis of variance (Kruskal-Wallis test). Results were considered significant at *p* value of less than 0.05.

## 3. Results

After considering the inclusion and exclusion criteria, 50 subjects (25 male, 25 female) were included in this study. The E group included 12 males and 13 females, while the C group included 13 males and 12 females. The average working time of the subjects was 8 hours per day. The baseline characteristics of the subjects are summarized in Table 1. At day 0, there were no significant differences in the age, pain, muscle endurance, and disability levels between the two groups (*p* > 0.05) (Table 1).

### 3.1. Pain

After 6 weeks of initiating the treatment, mean improvements in pain score in the E and C groups were 2.00 and 0.9 points, respectively. Compared to day 0, there was a statistically significant improvement in NP at days 14 and 42 in both groups (*p* < 0.05). On days 14 and 42, there were statistically significant differences in NPRS scores between the groups (*p* < 0.05) (Table 2).

### 3.2. Muscle Endurance

After 6 weeks of initiating the treatment, mean improvements in muscle endurance in the E and C groups were 5.26 and 2.83 mmHg, respectively. Compared with day 0, there were statistically significant improvements in muscle endurance at day 14 and day 42 in both groups (*p* < 0.05). At days 14 and 42, there were statistically significant differences in CCF test scores between the groups (*p* < 0.05) (Table 3).

### 3.3. Disability

After 6 weeks of initiating treatment, the mean improvements in NDI scores in the C and E groups were 6.77 and 2.78 points, respectively. Compared to day 0, there were statistically significant improvements in the disability on days 14 and 42 in both groups (*p* < 0.05). At day 42, there were statistically significant differences in the NDI scores between the groups (*p* < 0.05) (Table 4).

## 4. Discussion

This study was performed to determine the effects of pressure biofeedback training in addition to conventional exercises on pain, DCF muscle endurance, and functional disability in school teachers with NP. This study is a continuation of a previous research that was conducted to see the effect pressure biofeedback training on pain and disability in a similar population [10]. However, current study also included DCF muscle endurance as one of the outcome measures. There were no statistically significant differences in age, pain, muscle endurance, and disability between subjects in the two groups, showing their homogenous distribution. Although there was improvement in both groups, there was a statistically significant improvement in muscle endurance, pain, and disability in the subjects who underwent additional training with pressure biofeedback. These results should be further correlated clinically.

Improvements in pain in both groups can be explained by various mechanisms that have been reported to come into play following exercise therapy. These include, among others, an increase in endorphins, better neuromuscular control, and activation of muscle ergoreceptors, and subsequently, improvements in the disability have been shown to be associated with improvements in pain [10].

Improvement in muscle endurance or performance is concurrent with restoration of muscle balance with optimal flexibility of tight muscles and improved strength in weak and inhibited muscles [23]. In muscles, increased extensibility of muscles is linked with increased torque production [33]. Our results are in line with those of previous studies that demonstrated improvements in muscle endurance after DCF and scapular muscle training [30]. This improvement can also be explained on the basis of motor learning, which requires information from the external world as feedback. With biofeedback training, a patient is subjected to goal-oriented behavior, which can improve the motor behavior via reinforcement. DCF muscle training using pressure biofeedback provides external feedback regarding the performance of the task. It has been demonstrated that subjects can control the recruitment as well as discharge of motor units by auditory and visual feedback [34]. Similarly, in this study, we have demonstrated that by providing external visual feedback, the subjects could consciously improve their muscle endurance and performance.

It has also been reported that the physiological basis behind the increase in muscle strength is associated with the use of feedback training that could be due to an increase in the average firing rate, motor unit recruitment, and increased synchronization of the active motor unit [35]. Biofeedback, in any form, has been proposed to be a very useful intervention for musculoskeletal pain [36]. Improvements in cervical muscle endurance and strength have been shown to be concurrent with reduction in NP [37]. The results of this study simultaneously demonstrate reduction in NP and improvement of muscle endurance following biofeedback training.

With the increasing dependency on computers, mobile phones, and other forms of technology, NP has been reported to affect the young and older adults alike [10]. The prolonged use of such devices at work often leads to adoption of a poor neck posture, which is unconsciously maintained for a long time, leading to insufficiency in muscle performance during various activities [38]. Work-related pain develops over time and is caused either by work overload or by a poor working environment. Furthermore, its complex nature in teachers suggests that no single factor can be held solely responsible [39]. Anthropometric variations, stress, furniture in schools, and low colleague supports are some of the factors that may influence the development of pain [7].

Previous research has demonstrated that, in cases of NP, training of DCF muscles for its effective use is more effective than nonspecific strengthening of other neck muscles [29]. Our results also suggest that besides increasing muscle endurance, specific training of DCF muscles in addition to conventional exercises can also improve NP and functional disability.

Work-related musculoskeletal pain has been shown to affect performance and output [11]. Development of such symptoms at a relatively young age results in sick leaves or early retirement [10]. No single preventive or intervention strategy has been proposed to control NP in teachers.

### 4.1. Limitations

This study was done on a small sample size and did not follow-up for 12 weeks postintervention, which is becoming the standard follow-up time, due to time and financial constraints. Future studies with a larger sample size and longer follow-up period are warranted to elaborately investigate the effects of DCF muscle training using pressure biofeedback on NP and its duration, disability, cervical range of motion, and posture.

## 5. Conclusions

The results of this study demonstrate that additional use of pressure biofeedback was statistically significant in the reduction of pain and functional disability and the increase in muscle endurance in comparison with conventional exercises alone in teachers with NP. These results should be further correlated clinically. A dedicated time for exercises at school could help prevent the development of NP in teachers.

## Figures and Tables

**Table 1:** Baseline characteristics of the subjects.

	Experimental group (*n* = 25)	Control group (*n* = 25)	*p* value
Age, years	36.33 ± 6.01	36.45 ± 5.95	0.07
Pain, points	5.20 ± 0.99	5.40 ± 0.56	0.06
Muscle endurance, mmHg	23.58 ± 0.93	24.15 ± 0.95	0.05
Disability, points	16.22 ± 5.05	17.25 ± 5.65	0.05

The data are presented as mean ± standard deviation.

**Table 2:** Comparison of pain intensity (NPRS, points) between the groups: mean ± SD, *p* values.

		Experimental group (*n* = 25)		
		Day 0 5.20 ± 0.99	Day 14 4.56 ± 0.85	Day 42 3.20 ± 0.78
Control group (*n* = 25)	Day 0 5.40 ± 0.56	0.06	0.03^∗^	0.01^∗^
	Day 14 5.00 ± 0.64	0.06	0.04^∗^	0.02^∗^
	Day 42 4.50 ± 0.55	0.04^∗^	0.07	0.04^∗^

The data are presented as mean ± standard deviation. NPRS: numeric pain rating scale. Nonparametric analysis of variance (Kruskal-Wallis test). ^∗^Significant.

**Table 3:** Comparison of muscle endurance (mmHg) between the groups: mean ± SD, *p* values.

		Experimental group (*n* = 25)		
		Day 0 23.58 ± 0.93	Day 14 26.94 ± 1.25	Day 42 28.84 ± 0.98
Control group (*n* = 25)	Day 0 24.15 ± 0.95	0.05	0.04^∗^	0.03^∗^
	Day 14 25.92 ± 1.18	0.06	0.04^∗^	0.02^∗^
	Day 42 26.98 ± 0.95	0.04^∗^	0.09	0.04^∗^

The data are presented as mean ± standard deviation. Nonparametric analysis of variance (Kruskal-Wallis test). ^∗^Significant.

**Table 4:** Comparison of disability scores (NDI, points) between the groups: mean ± SD, *p* values.

		Experimental group (*n* = 25)		
		Day 0 15.22 ± 5.6	Day 14 11.85 ± 4.9	Day 42 8.45 ± 4.3
Control group (*n* = 25)	Day 0 13.50 ± 3.80	0.05	0.04^∗^	0.01^∗^
	Day 14 11.99 ± 3.68	0.01^∗^	0.05	0.01^∗^
	Day 42 10.72 ± 3.85	0.01^∗^	0.50	0.01^∗^

The data are presented as mean ± standard deviation. NDI: neck disability index. Nonparametric analysis of variance (Kruskal-Wallis test). ^∗^Significant *p* < 0.05.

## Data Availability

The datasets used in this study are available from the corresponding author on reasonable request.

## Abbreviations

NP: Neck pain
DCF: Deep cervical flexor
E group: Experimental group
C group: Control group
CCF: Craniocervical flexion
NPRS: Numerical pain rating scale
NDI: Neck disability index.
